# Role of Selexipag in Chronic Obstructive Pulmonary Disease (COPD) Patients With Out-of-Proportion Pulmonary Hypertension

**DOI:** 10.7759/cureus.16520

**Published:** 2021-07-20

**Authors:** Sherif T Abuserewa, Ahmed Selim, Amr Youssef, Ronald Zolty

**Affiliations:** 1 Internal Medicine, Mercer University School of Medicine, Grand Strand Health, Myrtle Beach, USA; 2 Department of Cardiovascular Medicine, University of Nebraska Medical Center, Omaha, USA

**Keywords:** copd: chronic obstructive pulmonary disease, out of proportion pulmonary hypertension, selexipag, copd exacerbation, exertional dyspnea, pulmonary hypertension

## Abstract

Pulmonary hypertension (PH) in patients with chronic obstructive pulmonary disease (COPD) is associated with an increase in the risk of COPD exacerbation, increased hospitalization, and worse survival in this patient population. No specific treatment is available for PH in COPD. However, reported out-of-proportion PH may benefit from a certain type of treatment. This study shows that the use of selexipag in the treatment of out-of-proportion PH in COPD patients was associated with an improvement in functional status evaluated by a six-minute walk test (6MWT) and a mean pulmonary artery pressure at 6 +/- 2 months of treatment.

## Introduction

Pulmonary hypertension (PH) is defined as an increase in the mean pulmonary artery pressure (mPAP) of ⩾ 20 mmHg at rest or >30 mmHg during exercise, which can complicate left heart and pulmonary diseases and result in right heart failure [1-2]. PH of a mild and moderate degree (mean PAP <35 mmHg) is a common complication of chronic obstructive pulmonary disease (COPD), which is mostly attributed to the remodeling of the pulmonary vasculature and is associated with an increased risk of COPD exacerbation and decreased survival [3]. COPD patients with "out-of-proportion" pulmonary - where resting mPAP was ≥ 35 mmHg in the presence of relatively preserved lung function hypertension - underwent several trials using pulmonary arterial hypertension (PAH)-directed therapy, including prostacyclin agonists, phosphodiesterase type 5 (PDE-5) inhibitors, and nitric oxide-cyclic guanosine monophosphate enhancers. These studies showed mixed effects on resting and exercise-induced pulmonary hemodynamics, six-minute walk test (6MWT), and quality of life [4].

Selexipag is a non-prostanoid prodrug metabolized in the liver to an active metabolite with a half-life of around eight hours. This metabolite has a very high affinity to the prostacyclin receptor (i.e., IP-receptor), one of five types of prostanoid receptors, which induces vasodilation and inhibits the proliferation of vascular smooth muscle cells. Selexipag was approved by the United States Food and Drug Administration (FDA) for the treatment of PAH to delay disease progression and reduce the risk of hospitalization [5-6]. In this study, we will evaluate the effect of selexipag on functional capacity in COPD patients with out-of-proportion pulmonary hypertension through the 6MWT and mean pulmonary artery pressure before and after selexipag treatment.

This study was presented as a poster presentation at the 23rd Annual Scientific Meeting of the Heart Failure Society of America in September 2019.

## Materials and methods

Study design

We performed a retrospective single-center case series observational study at the University of Nebraska Medical Center, Omaha, NE. We reviewed the medical records of COPD patients who were started on selexipag for out-of-proportion pulmonary hypertension. Six patients met the inclusion criteria and were included in this study for final analysis.

Patient population

Patients in this study were diagnosed with COPD according to the Global Initiative for Chronic Obstructive Lung Disease (GOLD) 2020 guidelines [7], with spirometry done within six months prior to the date of enrollment. All included patients had right heart catheterization showing out-of-proportion pulmonary hypertension with a resting mPAP of ≥ 35 mmHg in the presence of a relatively preserved lung function, i.e., less severe airflow limitation (mild to moderate obstruction with forced expiratory volume in the first second (FEV1) >50%), hypoxemia, normocapnia or hypocapnia, and exercise limitation of cardiovascular origin, i.e., no history of heart failure. The 6MWT and right heart catheterization were done at baseline and repeated after six months +/- two months of selexipag treatment.

Exclusion criteria included COPD patients in acute exacerbation or respiratory failure at the time of enrollment or severe or very severe obstructive pattern by spirometry, as well as patients with mPAP < 35 mmHg, history of structural heart disease or heart failure, with reduced ejection fraction (left ventricular ejection fraction < 50%). This study was approved by the University of Nebraska Institutional Review Board.

Spirometry

FEV1 and forced vital capacity (FVC) were obtained via spirometry within six months of right heart catheterization.

Right heart catheterization

Right heart catheterization was done to confirm the diagnosis and type of pulmonary hypertension at rest using the standard approach [8]. The patient was then exercised using arm lifting with increased resistance during the exercise test at a linear rate determined according to each patient’s prior exercise test performance or self-reported baseline fitness level, to aim for an eight to 12-minute symptom-limited test [9].

Vasoreactivity testing

Inhaled nitric oxide (iNO) at 10 to 20 ppm is the “gold standard” for pulmonary vasoreactivity testing [9-10]. Criteria for the definition of a pulmonary vasoreactivity response (VR) are a decrease in mean pulmonary artery pressure (mPAP) of ⩾ 10 mmHg to reach an absolute mPAP of < 40 mmHg [11].

## Results

Descriptive baseline characteristics of the included patients in the study are shown in Table 1.

**Table 1:** Patients' characteristics

Patients' characteristics	
Age (mean)	72
Sex	67% females
Race	100% White
Body mass index (BMI) (mean)	30.6
Obstructive sleep apnea (OSA)	67% have OSA
Hypertension (HTN)	83% Hypertensive
Diabetes mellitus (DM)	67% diabetics
Coronary artery disease (CAD)	60% have CAD
Pulmonary embolism (PE)	17% has PE
Smokers	67% smokers
Smoking packs/year (mean)	43
Forced expiratory volume in the first second (FEV1)/Forced vital capacity (FVC) (mean)	64
FEV1 (mean)	60%
FVC (mean)	71%

All included patients were of white ethnicity, with four out of six patients females (67%) and a mean age of 72 years. The patients’ BMI ranges differently, with two out of six in the overweight range, two out of six were in the mild obesity range, one out of six was in the moderate obesity range, and one out of six was in the normal BMI range. Moreover, different comorbidities were identified in these patients. Four out of six patients had OSA and DM, five out of six were hypertensive, three out of six had CAD, and only one of six had PE.

Of note, only four out of six were smokers with 43 mean smoking packs/year. Spirometry findings showed a mean FEV1/FVC of 64%, mean FEV1 of 60% (ranging from 51%-66%), and mean FVC of 71%.

We monitored the 6MWT, Borg scale, heart rate, oxygen saturation, and measurements of right heart catheterization (RHC) before and after six +/- two months after selexipag, and Table 2 shows the results.

**Table 2:** Results of monitoring Six-minute walk test (6MWT), Borg scale before/after 6MWT, heart rate before/after 6MWT, oxygen saturation before/after 6MWT, mean pulmonary artery pressure (mPAP), pulmonary vascular resistance (PVR), mean pulmonary capillary wedge pressure (PCWP), trans-pulmonary gradient (TPG), cardiac output, and cardiac index

	Results	
Mean readings	Before Treatment	After Treatment
6MWT	623	720
6MWT change	Improvement with 97 meters	
Borg scale before 6-MWT	2.5	1.5
Borg scale after 6MWT	3.6	3
Heart rate before 6MWT	83	79
Heart rate after 6MWT	96	104
Oxygen saturation before 6MWT	91	92
Oxygen saturation after 6MWT	88	88
RHC		
mPAP	49	39
PVR	7.5	4.7
Mean PCWP	14	18
TPG	35	21
Cardiac output	5.2	5.9
Cardiac index	2.7	3.1

As shown in Table 2, the Borg scale before and after 6MWT showed mild non-significant improvement after selexipag treatment. On the other hand, the heart rate and oxygen saturation haven’t shown any significant change.

**Figure 1:**
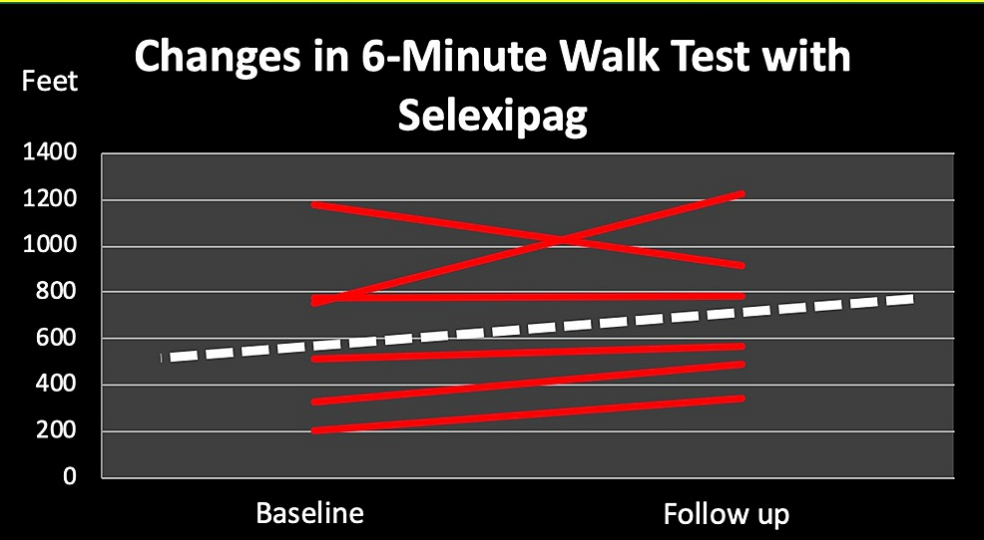
Changes in six-minute walk test over six months of selexipag treatment

The patient's functional status was assessed by 6MWT which has improved after treatment with selexipag with a total mean change of 97 meters. Out of the six patients included in this study, five patients showed improvement of the six-minute walk distance (from 512 feet to 681 feet, mean change 169 feet) while one patient worsened (from 1177 feet to 913 feet) as shown in Figure 1.

**Figure 2:**
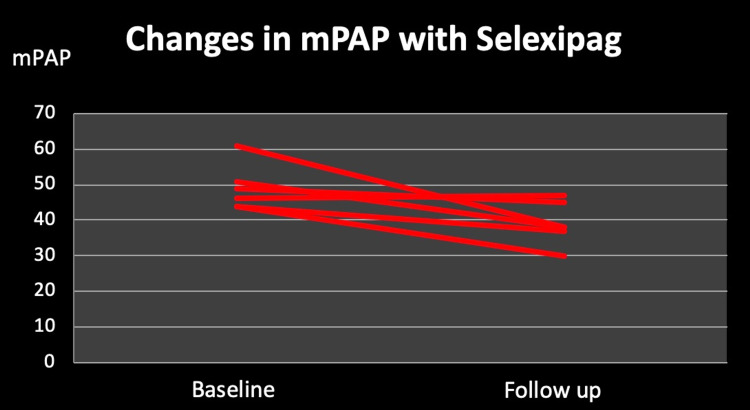
Changes in mean pulmonary artery pressure (mPAP) with selexipag treatment over six months

Furthermore, RHC findings showed significant improvement on follow-up after selexipag treatment in comparison to that before treatment. mPAP showed a significant decrease with a total mean change of 10 mmHg (49 mmHg to 39 mmHg). Out of the six patients included in this study, five patients showed a decrease in mPAP (from 50 mmHg to 38 mmHg, with a mean change of 12 mmHg) while one patient worsened (from 46 mmHg to 47 mmHg) as shown in Figure 2.

Moreover, pulmonary vascular resistance (PVR) also showed a significant decrease with a 2.8 Woods mean change (7.5 Woods to 4.7 Woods). Cardiac output and cardiac index improvement were also noticed on follow-up.

## Discussion

Pulmonary hypertension (PH) is a common complication of COPD although the actual overall prevalence of PH in COPD remains unclear [12-13]. The prevalence of PH in patients with COPD was shown to be around 70%, with only < 5% of them having severe PH [14-16]. Of note, all the patients included were in a stable COPD state and on long-term oxygen therapy (LTOT) because of unproportionate pulmonary hypertension and hypoxemia and optimal medical treatment. A subgroup of subjects with moderate to severe PH was identified as “out-of-proportion PH.” It is still unclear if the disproportionate PH should depend on airflow obstruction severity, specific cut-off values in hypoxia, certain vascular lesions, or different other parameters.

Since the introduction of LTOT, the survival of hypoxemic COPD patients has improved. However, pulmonary hypertension in COPD patients is associated with lower five-year survival, with mPAP reported to be a stronger predictor of poor outcomes compared to FEV1, hypoxemia, and hypercapnia [17]. Moreover, patients with mPAP >18 mmHg were showed to have a higher risk of severe COPD exacerbation, requiring hospital admission [18]. Patients with severe PH had pulmonary vascular obstruction and remodeling, resulting in low perfusion to alveoli and an increase in dead space, leading to a high V/Q mismatch. Although dead space in COPD patients with severe PH declines minimally at maximum exercise, the main limitation is circulatory and not ventilatory, so it might benefit from vasoactive medication [19].

Recent studies have shown that endothelial dysfunction plays an important role in the pathogenesis of PH in COPD [20-21]. However, data to support the use of PAH-specific therapies in patients with PH secondary to chronic parenchymal lung disease are limited. For example, a randomized study used Bosentan in the treatment of PH in COPD patients with stage III or IV and without severe PH at rest for 12 weeks resulted in worsening of hypoxemia [22] while in another non-randomized study, Bosentan showed significant functional improvement in those with COPD stage GOLD III or IV [23]. Furthermore, the use of sildenafil [24-26] and iloprost [27] in the same types of patients resulted in hemodynamic improvement without significant clinical benefit, but instead, it worsened hypoxia. However, it is important to mention that worsening hypoxia by PH-specific treatment was never of clinical significance.

According to the Seraphin [28] and Ambition trials results, a combination of a PDE5 inhibitor and either ambrisentan or macitentan is considered the current standard therapy for PAH [29]. However according to the GRIPHON study, on adding selexipag to the current standard therapy in comparison to the placebo, selexipag has significantly reduced the risk of all-cause mortality, hospitalization for worsening PAH, the need for lung transplantation, or balloon septostomy. It also significantly reduced the need for parenteral prostanoid therapy or long-term oxygen or disease progression as compared with placebo. The GRIPHON trial provides clear evidence of the ability of selexipag to reduce disease progression in all population subgroups and in the presence of optimal background therapy.

To our knowledge, this is the first study to evaluate and shed light on the effect of selexipag on out-of-proportion pulmonary hypertension. The study showed very promising results not only on the functional status of the included patients but also on the RHC findings in general and on mPAP in particular. However, there are several limitations to our study; the single-center retrospective nature of this case series is a major limitation. Also, all included patients were of white ethnicity with a female majority. These factors, as well as the small number of subjects included, might limit the generalizability to the rest of the population.

## Conclusions

In this case series of COPD patients with out-of-proportion pulmonary hypertension, the use of selexipag was associated with a trend towards improvement in patients’ functional status assessed by the six-minute walk test and reduction in mPAP at six months. However, larger studies are needed to better define the safety and efficacy of selexipag in this clinical setting.
